# Difficulty accessing contraceptives in a 2010–2022 prospective cohort of sex workers in Vancouver, Canada: intersectional influence of im/migration status and racialization

**DOI:** 10.1186/s12978-025-02214-8

**Published:** 2025-12-23

**Authors:** Emma Stirling-Cameron, Esteban Valencia, Kate Shannon, Haoxuan Zhou, Ran Hu, Grace Chong, Kaylee Ramage, Jennie Pearson, Shira Miriam Goldenberg

**Affiliations:** 1 School of Population and Public Health, University of British Columbia, Vancouver, BC Canada; 2 Faculty of Medicine, Division of Social Science, University of British Columbia, Vancouver, BC Canada; 3 Department of Statistics, Simon Fraser University, Burnaby, BC Canada; 4 College of Social Work, Ohio State University, Columbus, OH USA; 5 School of Public Health, University of Tennessee, Knoxville, TN USA; 6 Interdisciplinary Studies Program, University of British Columbia, Vancouver, BC Canada; 7 School of Public Health, San Diego State University, San Diego, CA USA

**Keywords:** Non-barrier contraceptives, Sex work, Migrants, Immigrants, Systemic racism, Canada

## Abstract

**Background:**

Overlapping and intersecting structural violence facing im/migrant sex workers has contributed to significant sexual health inequities, such as reduced odds of recent HIV testing, Sexually transmitted and blood borne illness treatment, and client condom refusal. Limited research has been conducted among im/migrant sex workers around access to contraception, particularly using an intersectional lens. The purpose of this paper is to examine the association between im/migration status and difficulty accessing non-barrier contraception among a prospective, community-based cohort of sex workers in Metro Vancouver, Canada, as well as whether this association is modified by racialization.

**Methods:**

Semi-annual questionnaire data were drawn from AESHA (*An Evaluation of Sex Workers Health Access*), an open, community-based longitudinal cohort of women sex workers in Vancouver, Canada (January 2010 – August 2022). We assessed the association between im/migration status and difficulty accessing non-barrier contraceptives (last 6 months), using racialization (Asian, Black, Latinx, or other racialized identity) as an effect modifier. We developed a multivariable confounder model using modified Poisson regression with generalized estimating equations using sandwich robust standard error.

**Results:**

Eight hundred and three participants were included, contributing a total of 5,133 observations over 12.8 years. The median number of visits per participant was four (range: 1–24). 32.6% of participants were im/migrants to Canada and 34.5% were Asian, Black, Latinx, or another racialized identity. In adjusted multivariable analysis exploring racialized identity as an effect modifier, participants who were both im/migrants and racialized faced the highest risk of experiencing difficulties accessing non-barrier contraceptives, when compared to non-im/migrant and non-racialized sex workers (Risk Ratio: 1.50; 95% CI: 0.99, 2.28).

**Conclusions:**

One-quarter of all participants reported experiencing difficulty accessing non-barrier contraceptives at least once during the study period. Im/migrant women sex workers who are Asian, Black, Latinx, or another racialized identity faced a greater risk of experiencing difficulties accessing non-barrier contraceptives compared to non-racialized, non-im/migrant sex workers. These findings indicate a critical need towards investment in culturally safe, linguistically congruent, and sex work-friendly sexual health services to increase contraceptive access and reduce the potential for sexual health inequities.

**Supplementary Information:**

The online version contains supplementary material available at 10.1186/s12978-025-02214-8.

## Plain Language Summary

This study aimed to understand how race and immigration status affect the ability of sex workers to access non-barrier contraception, such as birth control pills or intrauterine devices, in Metro Vancouver, Canada.

The research used data from a long-term study, AESHA* (An Evaluation of Sex Workers Health Access*), which followed women sex workers between 2010 to 2022. The study asked participants about their experiences accessing contraception over the past six months. It also looked at how race and immigration status affected their access, comparing the experiences of racialized immigrants (such as those who are Asian, Black, or Latinx) to those who were White and born in Canada.

The study involved 803 women who provided data for over 12 years, totaling more than 5,100 responses. About one-third of the women were immigrants, and just over a third were Asian, Black, Latinx, or another racialized identity. The results showed that sex workers who were both immigrants and racialized were more likely to have trouble accessing contraception compared to those who were White and born in Canada.

These findings highlight the need for better sexual health services that are culturally sensitive, language-appropriate, and welcoming to sex workers. Such services could help reduce barriers to contraception and improve sexual health outcomes for these groups, ultimately addressing health inequalities.

**Supplementary Information:**

The online version contains supplementary material available at 10.1186/s12978-025-02214-8.

## Introduction

In Canada and elsewhere, sex workers experience human rights violations and health and social inequities as a result of the continued stigmatization and criminalization of sex work. This includes a heightened risk of police- and client-perpetrated violence [1–3], sexually transmitted and blood borne infections (STBBIs) [4, 5], and occupational health and safety violations [6–8]. For sex workers who conduct penile-vaginal services, there is a disproportionate burden of unintended pregnancy [9] as a result of high rates of client condom refusal [6, 8, 10] and barriers to accessing non-barrier contraceptives (e.g., long-acting reversible contraception, short- and long-acting hormonal methods, and emergency contraception) [9]. Access to safe, non-stigmatizing sexual and reproductive health (SRH) services is paramount in supporting the occupational health and SRH of sex workers [6, 11]. In particular, access to and use of non-barrier contraceptives offers greater reproductive autonomy and greater protection against pregnancy than condoms alone [12–14]. Yet previous literature has shown that im/migrant (inclusive of refugee, asylum-seeking, immigrant, migrant and undocumented people) sex workers in Canada experience additional inequities regarding access and use of essential SRH services [15–17]. 

In the North American context, im/migrants, particularly racialized im/migrants from the Global South, are overrepresented within precarious labor industries, including sex work [18, 19]. Im/migrant women in Canada report diverse motivations for engagement in sex work, including exclusion from conventional labor opportunities due to language barriers, lack of acceptance of internationally obtained education, and racial and xenophobic discrimination, in addition to higher wages and more flexible working hours [18–21]. Current ‘end-demand’ Canadian sex work legislation (*the Protection of Communities and Exploited Persons Act*, PCEPA) criminalizes various aspects of the sex industry and explicitly prohibits migrants with temporary resident status (e.g., open work permit holders, international students) from selling sexual services [22]. This approach is not unique to Canada, with most other countries around the world criminalizing sex work in part or in whole [23]. Intersecting punitive sex work laws and prohibitive immigration policies have contributed to increased police surveillance and harassment, racial profiling, arrest and prosecution, loss of status, detainment, and/or deportation among im/migrant sex workers in Canada [20, 24–27]. 

The disproportionate surveillance and criminalization faced by im/migrant sex workers has been linked to barriers to SRH services and occupational safety concerns [3, 8, 24]. For example, qualitative research has demonstrated that frequent raiding of indoor sex work venues by immigration and police authorities and the use of condoms as evidence of criminalized activities can result in restricted access to HIV/STI testing and condoms for im/migrant sex workers by venue management [16]. Access to SRH for im/migrant sex workers is further compounded by structural marginalizations borne out of racism and xenophobia that impact care and safety for racialized and/or im/migrant woman [6, 28, 29]. This includes interpersonal racism from healthcare providers, differential health insurance provided to im/migrants in Canada or lack of extended coverage, and a lack of interpreters and interpretation services. The overlapping and intersecting structural violence facing im/migrant sex workers has contributed to significant SRH inequities, such as reduced odds of recent HIV/STI testing [17], and reports of self-medication for STBBI treatment [6], yet we know little about impacts on broader SRH services such as access to non-barrier contraception.

The landscape of contraceptive care has changed dramatically over the past decade in the Canadian province of British Columbia. Prior to 2023, patients wanting to access non-barrier contraceptives required a prescription from a licensed healthcare provider (i.e., physician, nurse practitioner) which is presented to a pharmacy for dispensation. The cost of prescription contraception must be paid for out-of-pocket or through an extended health benefits insurance plan through a private insurer. Recent improvements to accessibility have included pharmacist prescribing rights (June, 2023) [30], and a recent decision to provide select contraceptives at no charge as part of the provincial PharmaCare program (BC’s publicly funded pharmacare program that helps B.C. residents pay for some prescription drugs; April 1, 2023) [31]. Yet contraceptive care for sex workers, particularly im/migrant sex workers, often looks different. New PharmaCare coverage only extends to individuals with provincial health care coverage [BC Medical Services Plan], which excludes select im/migrant populations, such as asylum-seekers, undocumented people, and those with work and study permits [32]. The self-employed and precarious nature of sex work under Canadian legislation means most workers do not have extended health benefits to cover the cost of prescription drugs. Sex workers in Canada have further reported anti-sex work discrimination from healthcare providers, including family physicians, who remain the most prominent providers of birth control prescriptions in the province [33, 34]. 

Intersectional Theory [35], pioneered by Kimberlé Crenshaw and other Black feminist scholars, is a framework used for understanding how overlapping social identities—such as race, gender, socioeconomic status, and im/migration status—interact with systems of power and oppression to influence health and social inequity. Intersectionality informs us as to how those facing multiple axes of oppression may experience unique challenges when seeking healthcare services and supports, and how this may influence health inequities and disparities in access. Previous literature exploring access to contraception has largely looked at the experiences of racialized women, sex workers, and im/migrant women in silo [9, 36, 37], failing to examine the experiences of women who may belong to multiple marginalized identities. Moreover, literature addressing contraceptive access among sex workers of all identities remains limited, with existing research largely focusing on male condom use [8, 10, 16, 38]. A recent systematic review of sex workers contraceptive access in North America only identified two large epidemiologic studies, with no specific additional attention given to racialization or im/migration status [39]. Thus, the purpose of this paper is to examine the association between im/migration status and difficulty accessing non-barrier contraception among a prospective, community-based cohort of sex workers in Metro Vancouver, Canada, as well as whether this association is modified by racialization.

## Methods

### Study design

Longitudinal data were drawn from an open, prospective, community-based cohort of sex workers, *An Evaluation of Sex Workers Health Access (AESHA). *Recruitment began in January 2010 and ended in June 2024. As previously described [40], AESHA was developed in conjunction with community sex work advocacy organizations and continues to be monitored by a large Community Advisory Board with representatives from diverse sex work and community organizations. Time-location sampling was used to recruit sex workers through daytime and late-night outreach in outdoor/public sex workspaces (e.g., alleyways, streets) and indoor venues (e.g., massage parlours, micro brothels) across Metro Vancouver (i.e., Surrey, Richmond, Burnaby, New Westminster). Locations for recruitment were identified through community mapping done with current and former sex workers and was updated regularly. Online recruitment was also used to reach sex workers engaged in online sex work and/or online solicitation spaces. Participants provided written informed consent prior to enrollment.

Trained interviewers with extensive community experience and/or lived experience as a sex worker administered questionnaires to participants at baseline and every six months after. Interviews were conducted in English, Cantonese, or Mandarin, and collected data on a range of sociodemographic, mental health and substance use, social and inter-personal, and structural characteristics, which have been described elsewhere [40]. Additionally, voluntary testing and pre- and post-test counseling is provided by a research nurse for STBBIs (i.e., HIV, syphilis, chlamydia, gonorrhea, Hepatitis C, HSV). Participants are offered treatment onsite and receive active referrals to health, clinical, and other services as needed. At inception (January 2010), participants received a $40 honorarium for each baseline or semi-annual follow-up visit. From September 2021 to March 2022, this amount was adjusted to $65 per visit, given the increased burden to participants of revised questionnaires which were expanded to address impacts of the COVID-19 pandemic. In May 2022, this was further increased to $80 per baseline visit and $65 per semi-annual follow up visit, to account for inflation and participant burden related to questionnaire length. The study has been approved by Providence Health Care/University of British Columbia’s Research Ethics Board.

### Study population

Inclusion criteria at baseline included identifying as a woman/femme while working, having exchanged sex for money within the past 30 days, were aged 14 years or older, and could provide informed consent. Our recruitment criteria are inclusive of diverse and fluid identities while capturing the ways that patriarchal gender norms shape participants’ experiences in sex work. Eligibility is inclusive of cis and transgender women, transexual women, and other transfeminine identities at enrolment. For the purposes of this analysis, we restricted the study population to people of reproductive age (18–49) who had never undergone a hysterectomy or tubal ligation. Altogether, the study sample included 803 participants who contributed 5,133 observations over 12.8 years. The median number of visits per participant was four (range: 1–24). The median time elapsed between participant visits was 6 months (range: 0–120 months).

### Outcome, exposure, and effect measure modifier

The outcome of interest was a binary indicator that reflected whether a participant experienced any difficulties accessing non-barrier contraceptives within the last six months. This indicator was derived from responses to the question: “In the last 6 months, what barriers to receiving contraceptives (aside from condoms) have you experienced?” Participants could select multiple barriers across different domains. Table 1 reports these domains and their descriptions, as presented to participants. Participants who responded affirmatively to any of the domains were classified as *experienced difficulties accessing non-barrier contraception in the last six months*. While participants could report experiencing multiple barriers, the distribution of multiple responses at any given study visit was sparse, which motivated the binary operationalization of the outcome.

**Table 1:** Classification of difficulties accessing non-barrier contraception by response domains

Domain	Description
Approachability	Barriers related to lack of health service information, e.g., don’t know where to go, don’t know what’s available, assume no services available
Acceptability	Barriers related to cultural and social factors, e.g., can’t get a doctor of preferred ethnicity or culture or gender, care that doesn’t meet the needs according to culture or gender, language barriers
Availability	Barriers related to accessing a healthcare setting, connecting with a healthcare provider, getting healthcare services in a timely manner, e.g., distance or transportation issues, inaccessible location for wheelchairs or strollers, hard to get appointment, limited hours of operation or services, long wait times, required to have multiple appointments
Affordability	Barriers related to cost of services, e.g., cost, health insurance barriers, have to miss work or arrange for childcare
Appropriateness	Barriers related to the gap between services and clients’ health needs, particularly on an interpersonal level unrelated to social/cultural factors, e.g., relationship with doctor, issues with treatment by health care professionals or staff, provider having enough time to talk to you, comfort level discussing contraceptives with provider, past negative experiences, empowerment, shared decision-making, trust
Stigma	Barriers rooted in stigma related to HIV status, race, sexual orientation or gender identity, cultural factors, sex work, drug use, or other factors
Other	Open text box for participants to discuss barriers not listed above, e.g., side effect profiles of non-barrier contraceptives, family doctor shortage, struggling with substance use

Our primary exposure of interest was *im/migrant status* (binary variable). Participants born in Canada were coded as *non-im/migrants*, and those born outside of Canada, regardless of im/migration timing or status, were coded as *im/migrants*. This coding captured a range of im/migrant identities, including immigrants and those who many have irregular or temporary status (e.g., resettled refugees, refugee claimants, immigrants, undocumented people, international students). Our effect modifying variable was *racialization* (binary variable). Participants who identified as Asian, Black, Latinx, or another racialized identity were coded as racialized, with white participants coded as non-racialized. Racial identities were collapsed into a single binary variable due to small cell counts in select racial groups. The term *racialized* refers to the process by which individuals or groups are socially constructed, categorized, and treated as belonging to a particular race, often resulting in unequal power dynamics and discrimination rooted in white supremacy. Exploring racialization as a potential effect modifier of the association between im/migration status and difficulty accessing non-barrier contraception among sex workers will allow for examination of associations across individual and intersecting identities.

### Covariates

We considered the following individual- and structural-level factors as a priori confounders based on existing literature: age in years, injection drug use in the last six months, unstable housing in the last six months, and inconsistent condom use for penetrative sex with one-time clients in the last six months. Covariates were treated as time-varying binary indicators (yes v/s no) except for age, which was treated continuously.

Descriptive analyses explored additional individual and structural factors, including: minority gender identity (e.g., transgender, non-binary, intersex, gender fluid, genderqueer, and Two-Spirit), minority sexual identity (e.g., Two-Spirit, lesbian, gay, bisexual, queer, asexual), timing of im/migration to Canada, citizenship status, discomfort speaking English, years engaged in sex work, primary location for soliciting and for servicing clients, and whether participants graduated high school.

### Data analysis

Descriptive analyses compared individual- and structural-level covariates at baseline stratified by the outcome. We compared categorical variables using Fisher’s exact test and compared continuous variables using the Wilcoxon rank-sum test. Modified Poisson regression modeled bivariate associations between individual- and structural-level factors and the outcome of difficulty accessing non-barrier contraceptives in the last six months [41, 42]. 

Primary analyses assessed whether racialization modified the association between im/migration status and difficulties accessing non-barrier contraceptives. Bivariate and multivariable modified Poisson regression estimated risk ratios (RRs) and 95% confidence intervals (95% CIs) [41, 42]. Following the recommendations of Knol and VanderWeele (2012) [43], we evaluated the presence of effect measure modification related to im/migration and racialization using four approaches: (1) re-parameterizing the exposure and modifier into a single 4-level categorical variable with a common referent, (2) examining the exposure-outcome association stratified by levels of the modifier, (3) modeling effect modification using a multiplicative interaction term, and (4) calculating the relative excess risk due to interaction (RERI) and accompanying 95% CI [44] to assess for additive effect modification.

All regression models accommodated the longitudinal structure of the data by using generalized estimating equations (GEE) with an exchangeable correlation matrix. All analyses used complete case observations; primary multivariable analyses removed 120 incomplete observations (2.3% of the total study population). All analyses were performed in R using version 4.3.3 [45] and all p-values are two-sided.

## Results

**Fig. 1:**
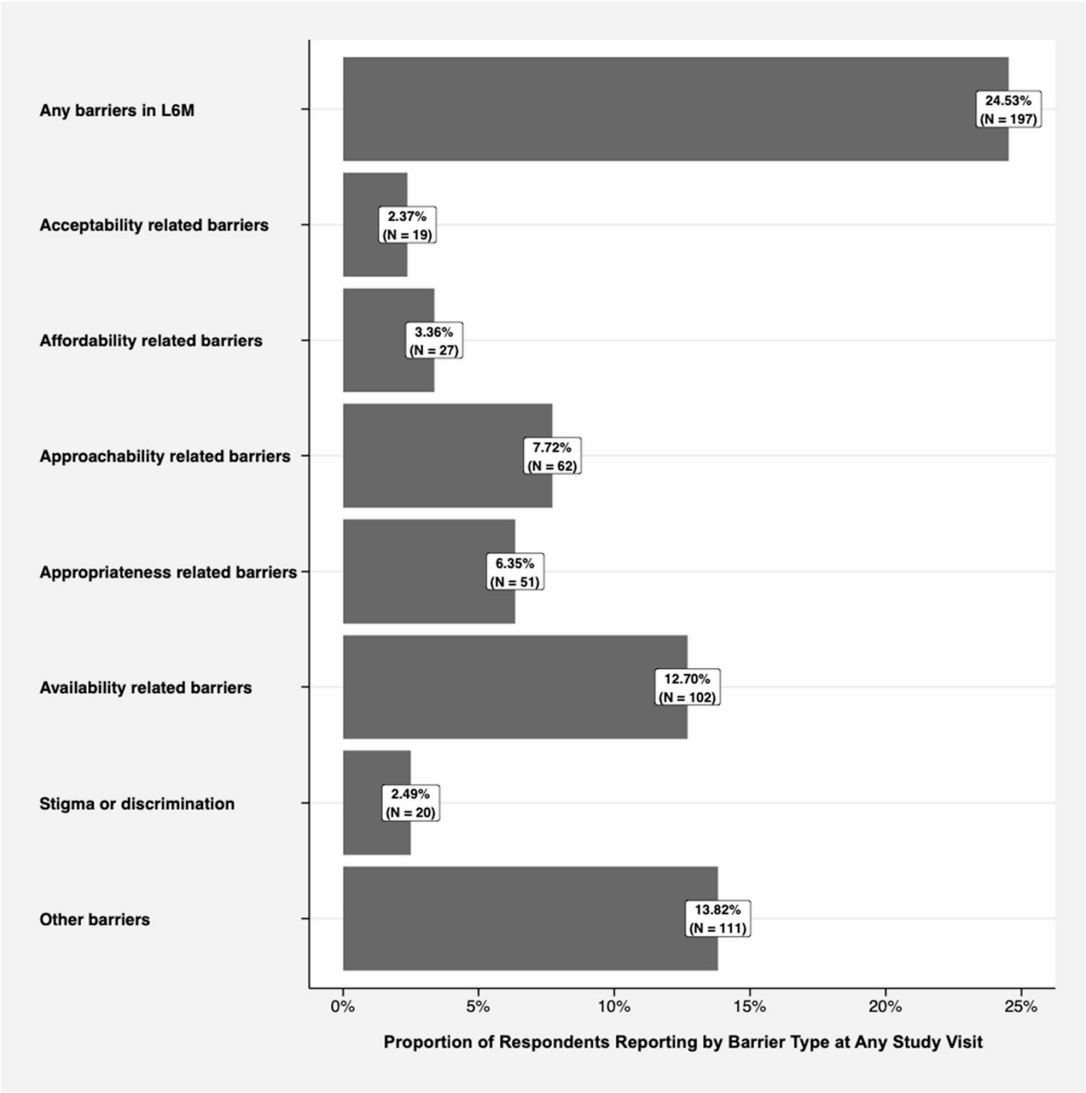
Frequency Distribution of Contraceptive Barriers Experienced By Sex Workers in Metro Vancouver in Last 6 Months, stratified by study period, 2010–2022 (*N* = 803). *Note: Participants may have selected multiple response options; as such, the sum of barriers experienced exceeds 197 (the total number of respondents who reported experiencing barriers to contraceptive access)

Eight hundred three respondents contributed 5,133 observations during the 12.8-year study period. Baseline characteristics of the sample stratified by difficulty accessing non-barrier contraceptives among our sample are reported in Table 2. Over the study period, 24.5% of participants (*n* = 197) reported experiencing difficulties accessing non-barrier contraceptives. Among those who reported any barriers to accessing contraception at any given study visit in the last six months: 194 reported experiencing 1–2 difficulties (3.78% of total observations), 104 reported experiencing 3–4 difficulties (2.02%), and 13 reported experiencing 5 or more difficulties (0.10%). Most frequently reported barriers to contraceptive use were related to availability (12.7%), approachability (7.7%), and appropriateness (6.3%; see Fig. 1). Median age at baseline was 33 years, 34.4% identified as racialized, 32.5% were im/migrants (12.9% as recent and 17.9% as long-term), and 12.1% did not have Canadian citizenship. Participants had been engaged in sex work for a median of 8 years. Participants were primarily soliciting clients in outdoor/public spaces (45.3%), followed by indoor spaces (informal or formal, including, apartments, hotels, massage parlours; 33.5%). 39.4% of participants used injection drugs and 70.9% reported unstable housing. In bivariate GEE analysis, non-citizens, those who are older, and those working longer in sex work faced an elevated risk of experiencing difficulty accessing non-barrier contraceptives (Table 3).

**Table 2:** Baseline characteristics stratified by difficulty accessing non-barrier contraceptives among sex workers in metro Vancouver, 2010–2022 (*N* = 803)

Characteristics	Total Affirmative Response (%) (*n* = 803)	Difficulty accessing non-barrier contraception		*p*-value
		Yes (%) (*n* = 43)	No (%) (*n* = 760)	
Individual factors				
Age (median, IQR)	33 (27–40)	30 (27–40)	33 (27–40)	0.438
Sexual Minority	303 (38.0)	19 (44.2)	284 (37.7)	0.392
Gender Minority^†††^	78 (9.7)	NS	73 (9.6)	0.599
Racialization (Yes v/s No)	276 (34.4)	19 (44.2)	257 (33.8)	0.164
Racialization (Categorical)				0.132
White (ref)	247 (30.8)	15 (34.9)	232 (30.5)	
Indigenous	280 (34.9)	NS	271 (35.7)	
Asian, Black, Latinx, other racialized identity ^††^	276 (34.4)	19 (44.2)	257 (33.8)	
Im/migration factors				
Im/migrant to Canada	261 (32.5)	19 (44.2)	242 (31.8)	0.093
Im/migration timing				0.171
Non-im/migrant	542 (69.2)	24 (58.5)	518 (69.8)	
Recent im/migrant (< = 5 years) ^†††^	101 (12.9)	NS	92 (12.4)	
Long-term im/migrant (> 5 years) ^†††^	140 (17.9)	NS	132 (17.8)	
Not a Canadian Citizen	81 (12.1)	11 (28.9)	70 (11.1)	0.003
Not comfortable speaking English	234 (29.3)	15 (34.9)	219 (28.9)	0.404
Sex work factors				
Years engaged in sex work (median, IQR)	8 (2–16)	5 (1–13)	8 (2–16)	0.088
Primary place soliciting clients^†^				0.118
Street/public (ref)	359 (45.3)	13 (30.2)	346 (46.1)	
Indoor (informal or formal)	266 (33.5)	16 (37.2)	250 (33.3)	
Independent	167 (21.1)	14 (32.6)	153 (20.4)	
Not engaged in sex work^†††^	NS	NS	NS	
Primary place servicing clients^†^				0.494
Outdoor/public (ref)	312 (39.7)	14 (32.6)	298 (40.1)	
Informal indoor	198 (25.2)	14 (32.6)	184 (24.8)	
Formal indoor	275 (35.0)	15 (34.9)	260 (35.0)	
No sex work^†††^	NS	NS	NS	
Inconsistent condom use for vaginal/anal sex with clients^†^	180 (22.6)	11 (26.2)	169 (22.4)	0.572
Sociostructural factors				
Any injection drug use^†^	316 (39.4)	19 (44.2)	297 (39.1)	0.509
Any unstable housing^†^	568 (70.9)	28 (65.1)	540 (71.2)	0.390
High school graduate	450 (56.1)	22 (51.2)	428 (56.4)	0.502

All data refer to *n* (%) of participants, unless otherwise specified

NS: *n* < 10 is not reported for confidentiality purposes

† Time-updated variable with recall period of the last 6 months

†† Racial identities collapsed due to low response numbers in particular groups

††† Number of observations suppressed when less than 10 (indicated by NS)

**Table 3:** Bivariate and multivariate analysis of factors associated with difficulty accessing non-barrier contraceptives among sex workers, 2010–2022 (*N* = 801)

Characteristic	Risk Ratio (RR) (95% CI)	*p*-value
Individual factors		
Age	0.972 (0.956, 0.988)	0.001
Sexual Minority	1.069 (0.819, 1.396)	0.623
Gender Minority	0.746 (0.464, 1.200)	0.227
Asian, Black, Latinx, other racialized identity (Yes v/s No)	1.126 (0.828, 1.530)	0.449
Race (Categorical)		
White	Reference	--
Indigenous	0.794 (0.583, 1.083)	0.146
Asian, Black, Latinx, other racialized identity^††^	0.992 (0.694, 1.418)	0.966
Im/migration factors		
Im/migrant to Canada	1.151 (0.846, 1.566)	0.371
Im/migration timing		
Non-im/migrant	Reference	--
Recent im/migrant (< = 5 years)	1.586 (0.987, 2.551)	0.057
Long-term im/migrant (< 5 years)	0.924 (0.629, 1.358)	0.687
Not a Canadian citizen	1.507 (1.009, 2.251)	0.045
Not comfortable speaking English	1.016 (0.727, 1.419)	0.927
Sex work factors		
Years engaged in sex work	0.983 (0.970, 0.996)	0.013
Primary place soliciting clients^†^		
Street/public	Reference	--
Indoor (informal or formal)	0.970 (0.676, 1.393)	0.870
Independent	1.127 (0.837, 1.517)	0.430
No sex work	0.944 (0.697, 1.279)	0.712
Primary place servicing clients^†^		
Outdoor/public	Reference	--
Informal indoor	1.142 (0.849, 1.537)	0.381
Formal indoor	0.996 (0.681, 1.457)	0.985
No sex work	0.957 (0.696, 1.317)	0.712
Inconsistent condom use for vaginal/anal sex with clients ^†^	1.399 (1.052, 1.860)	0.021
Sociostructural factors		
Any injection drug use^†^	1.128 (0.871, 1.461)	0.361
Any unstable housing^†^	1.093 (0.812, 1.471)	0.559
High school graduate	0.844 (0.643, 1.108)	0.222

Table 4 reports the findings our effect modification analysis, assessing whether risk of experiencing difficulty accessing non-barrier contraception by im/migration status was modified by racialization. We observed a gradient in the risk of difficulty accessing non-barrier contraception among the intersections of im/migration and racialization, with the highest risk of difficulty accessing non-barrier contraception observed among im/migrant women who are Asian, Black, Latinx, or another racialized identity (RR: 1.498; 95% CI: 0.986, 2.275), followed by im/migrant women who are not racially minoritized (RR:1.372; 95% CI: 0.714, 2.638), and Asian, Black, Latinx, or another racialized identity who are not im/migrants (RR: 1.098; 95% CI: 0.551, 2.186), when compared to people who are neither im/migrants or racially minoritized. Within strata of racialization, similar RRs were reported across groups. There was no observed additive interaction (RERI: 0.03, 95% CI: −1.21, 1.26), indicating that the joint effect of racialization and im/migration status is not greater than the sum of their individual effects.

**Table 4:** Effect modification of the association between im/migration status and difficulty accessing contraceptives by racialization, 2010–2022 (*N* = 801)

Outcome: Difficulties accessing non-barrier contraceptives in last 6 months				
		Asian, Black, Latinx, other racialized identity		
		Yes (*n* = 555)	No (*n* = 246)	Risk Ratio (RR) (95% CI) for im/migration status within strata of racialization
		RR (95% CI)	RR (95% CI)	
Im/migrant	Yes (*n* = 261)	1.498 (0.986, 2.275)	1.372 (0.714, 2.638)	1.098 (0.551, 2.186)
	No (*n* = 540)	1.098 (0.551, 2.186)	Reference	1.092 (0.528, 2.256)

Measure of interaction on additive scale: RERI (95% CI) = 0.028 (−1.207, 1.264)

Measure of interaction on multiplicative scale: multiplicative interaction term, RR (95% CI) = 0.995 (0.375, 2.641)

Multivariable models estimated by modified Poisson regression using generalized estimating equations (GEE), adjusted for age, injection drug use, unstable housing, and inconsistent condom use

*RR* Risk ratio

## Discussion

In this 12-year community-based cohort study of women sex workers, approximately one-quarter of all participants reported experiencing difficulty accessing non-barrier contraceptives at least once during the study period. Effect modification analyses found that im/migrant women sex workers who are Asian, Black, Latinx, or another racialized identity, faced a greater risk of experiencing difficulties accessing non-barrier contraceptives compared to non-racialized, non-im/migrant sex workers. Among all im/migrant sex workers, only those who were also racialized had a marginally significant risk, with a gradient in risk observed from non-racialized, non-im/migrant women to racialized im/migrant women. Bivariate comparisons also suggest that other im/migration-related indicators, such as lacking citizenship and im/migration recency, were associated with difficulty accessing non-barrier contraceptives.

Results from our study affirm the growing body of international evidence that has documented gaps in access to non-barrier contraceptives for sex workers [9, 46–50], and builds upon this by utilizing an intersectional approach to examine the added risk faced by racialized im/migrants when compared to non-racialized, non-im/migrant sex workers. For example, a global systematic review on STBBI prevalence and sexual health service access among im/migrant sex workers identified precarious im/migration status as a structural factor associated with a heightened burden of HIV, syphilis, and hepatitis C among recent im/migrant sex workers [15]. Our findings take this further, with results indicating the importance of the intersectional impact of im/migrant status and racialization on difficulty accessing contraceptives, rather than im/migration alone. Similar findings were recently reported by Hu et al. (2023) which found that Asian sex workers without Canadian citizenship faced significantly reduced odds of accessing health services when needed. Across both studies the interaction of multiple structural marginalizations—which are frequent among im/migrant sex workers in the Canadian context—was associated with greater risk of experiencing inequitable access to care.

This and other studies, largely in the Canadian context, have attributed SRH inequities with the continued criminalization of sex work. In Canada, many im/migrant populations are explicitly prohibited from engaging in sex work [21]. Among im/migrant sex workers in Canada, fear of immigration and law enforcement surveillance, potential for arrest and detainment, loss of im/migration status, and potential for deportation are pervasive barriers to SRH service access [15, 16, 18, 20]. In the Canadian context, the over-policing and frequent raiding of formal indoor venues (e.g., massage parlous) by law enforcement has contributed to restricted access to condoms, sexual health education, and STBBI testing onsite, fearing they may be used as evidence [16, 18]. 

These barriers, rooted in criminalization of sex work, are experienced in tandem with other systemic, institutional, and interpersonal factors liable to affect any racialized and/or im/migrant woman in a predominantly white host nation. The exclusion of certain im/migrants from public health insurance, limited access to linguistically, culturally, and/or racially congruent SRH providers, difficulty navigating siloed health services, and systemic and interpersonal racism and/or xenophobia have the potential to additionally influence SRH access for racialized im/migrant sex workers [15, 51]. Further mixed-methods or qualitative work is urgently needed to more definitely identify contributing factors to difficulties accessing contraceptives for racialized im/migrant sex workers in Canada in order to better inform future interventions and/or policy changes.

Although the province of British Columbia took welcome strides to reduce insurance-related difficulties to accessing non-barrier contraceptives in 2023, gaps remain for im/migrants, which must be addressed to advance reproductive equity. As of April 1, 2023, BC PharmaCare (British Columbia’s publicly funded program that helps B.C. residents pay for some prescription drugs) assumed the cost of select contraceptives, including certain hormonal and copper intrauterine devices, hormonal birth control pills, emergency contraceptives, subdermal implants, and injectables for all persons enrolled in BC’s public health insurance (BC Medical Services Plan; BC MSP) [31]. A similar structure is intended to rollout across Canada in 2025 [52]. Unfortunately, many im/migrants, particularly asylum-seekers, undocumented people, and those with work and study permits, are not eligible for BC MSP or receive differential coverage, necessitating out-of-pocket payments to access contraceptives [53]. Despite the intent of this policy to provide ‘universal contraceptive coverage,’ a key population has been neglected from this initiative, and are liable to continue to face significant financial barriers to accessing contraception. This study further confirms the need to expand contraceptive coverage for all residents, regardless of im/migration status, as has already been advocated for by community organizations in Vancouver, BC [32].

### Strengths and limitations

To our knowledge this is the first epidemiologic study in North America to describe non-barrier contraceptive access among sex workers using an intersectional lens, particularly with regards to im/migration status. However, our findings should be contextualized within a few major limitations. First, all study data were self-reported and may be subject to information bias. Yet data were collected by multilingual staff with lived or living experience as sex workers, who built rapport with participants through ongoing outreach, mitigating sources of information bias (namely, recall and social desirability bias). Second, the distribution of repeated observations across study participants was unbalanced due to the open-cohort design of the AESHA project. We explore this issue further in Supplement A but acknowledge that the open-cohort design could vulnerate our study to cohort effects, whereby findings reflect the experiences of women who joined AESHA in its earlier years while masking experiences of recent participants.

Ultimately, we were unable to address this issue analytically and explored it only descriptively. Given the marginalizations often experienced by the study population, and high rates of mobility, an open cohort design is most feasible for longitudinal data collection, and a community-oriented approach. Third, we operationalized our outcome as a binary indicator of barriers accessing contraception. While data were available on the frequency and type of barriers women recently encountered, our decision to use a binary outcome indicator was informed by the sparse distribution of multiple barriers in the last six months (Supplement B) and the lack of theoretical foundation for categorizing barriers based on severity. Thus, we make no inferences about how racialization and immigration status affect the severity and frequency of difficulty accessin contraception. Fourth, while AESHA regularly collected data on self-reported gender identity, data on sex assigned at birth was only collected as of 2021, resulting in significant missingness across the study period. Accordingly, our study population included participants who were not capable of becoming pregnant because they were assigned male at birth (AMAB). However, among valid responses to questions regarding sex assigned at birth, approximately 2% of participants self-reported as AMAB. Because of this, we conclude that the impact of including AMAB participants in the study population was likely negligible; further, while participants of minoritized gender identities comprised approximately 10% of the overall study population, we recognize this primarily reflects the experiences of participants who were assigned female at birth but who do not identify as cisgender female. Finally, this study was limited in the availability of data on specific sociocultural barriers and facilitators to contraceptive access, future research evaluating these and which informs potential interventions to increase culturally appropriate and voluntary access to non-barrier contraceptives for racialized im/migrant sex workers is recommended.

## Conclusion and recommendations

With 12 years of longitudinal data, we found racialized im/migrant women to be at a 1.5 times increased risk of facing difficulties accessing contraception when compared to non-racialized, Canadian-born women. These findings indicate a critical need towards investment in culturally-safe, linguistically congruent, and sex work-friendly sexual and reproductive health services. Broader efforts to address systemic racism and the criminalization of im/migrant sex workers are urgently needed in the criminal-legal and healthcare system. Additional policy reforms must be advocated for, including the complete decriminalization of sex work for all [18, 20] and expansion of public health insurance for im/migrant populations currently denied enrollment in Medical Insurance Plans across Canada.

## Supplementary Information

Supplementary Material 1.

## Data Availability

Data for this study are not publicly available for legal and ethical reasons, as this study involves sensitive data collected with a highly criminalized and stigmatized population of marginalized women. Under our current ethical approvals by the Providence Health Care – University of British Columbia (PHC-UBC) Institutional Research Ethics Board, de-identified data can be made available upon reasonable request and pending ethical approval. Please submit all requests to initiate the data access process to the corresponding author and PHC-UBC REB at [ubc.all-reb@ubc.ca] (mailto: ubc.all-reb@ubc.ca).

## Abbreviations

STBBI: Sexually transmitted and blood borne infection
Im/migrant: Person of any status who was born outside of Canada, inclusive of immigrants, migrants, refugees, asylum seekers, undocumented people
GEE: Generalized estimating equations
RR: Risk Ratio
IUD: Intrauterine device
PCEPA: Protected Communities and Exploited Persons Act
STI: Sexually transmitted infection
HIV: Human immunodeficiency virus
