# Supplementary material for: Difficulty accessing contraceptives in a 2010–2022 prospective cohort of sex workers in Vancouver, Canada: intersectional influence of im/migration status and racialization
Source: Reprod Health. 2025 Dec 23;23:19. doi: 10.1186/s12978-025-02214-8 (PMC12849248; doi:10.1186/s12978-025-02214-8)
Supplement: Supplementary file 1 — Supplementary Material 1. [file 12978_2025_2214_MOESM1_ESM.docx]

**SUPPLEMENT A - Analysis of Study Participants by Duration of Participation**

Figure A.1: Distribution of Study Participation Among Sex Workers in Metro Vancouver, 2010- 2022 (N=803)

Table A.2: Baseline Characteristics of Sex Workers in Metro Vancouver by Duration of Participation, 2010- 2022 (N=803)

| **Characteristic** | Overall  (N = 803) | **Total Number of Observations per Participant** | | | | **p-value^2^** |
| --- | --- | --- | --- | --- | --- | --- |
|  |  | 1 or 2  (N = 307) | 3 or 4  (N = 111) | 5 or 6  (N = 90) | 7 or more  (N = 295) |  |
| **Individual Factors** |  |  |  |  |  |  |
| Age^1^ | 33 (27, 40) | 33 (27, 40) | 34 (27, 41) | 35 (28, 41) | 32 (27, 40) | 0.250 |
| Sexual Minority | 43 (5.35) | 17 (5.54) | 9 (8.11) | 7 (7.78) | 10 (3.39) | 0.140 |
| Gender Minority | 303 (38.02) | 82 (27.24) | 28 (25.23) | 36 (40.00) | 157 (53.22) | <0.001 |
| Racialized (Yes v/s No) | 276 (34.37) | 150 (48.86) | 47 (42.34) | 38 (42.22) | 41 (13.90) | <0.001 |
| Racialized (Categorical) |  |  |  |  |  | <0.001 |
| White (reference) | 247 (30.76) | 79 (25.73) | 39 (35.14) | 29 (32.22) | 100 (33.90) |  |
| Indigenous | 280 (34.87) | 78 (25.41) | 25 (22.52) | 23 (25.56) | 154 (52.20) |  |
| Asian, Black, Latinx, or Other Racialized Identity | 276 (34.38) | 150 (48.86) | 47 (42.34) | 38 (42.22) | 41 (13.89) |  |
| **Im/migration Factors** |  |  |  |  |  |  |
| Im/migrant to Canada | 261 (32.50) | 141 (45.93) | 44 (39.64) | 36 (40.00) | 40 (13.56) | <0.001 |
| Im/migration Timing |  |  |  |  |  | <0.001 |
| Non-Im/migrant | 542 (69.22) | 166 (56.27) | 67 (61.47) | 54 (62.79) | 255 (87.03) |  |
| Recent Im/migrant (< 5 years) | 101 (12.90) | 57 (19.32) | 15 (13.76) | 17 (19.77) | 12 (4.10) |  |
| Long-term Im/migrant (> 5 years) | 140 (17.88) | 72 (24.41) | 27 (24.77) | 15 (17.44) | 26 (8.87) |  |
| Not a Canadian Citizen | 81 (12.09) | 35 (15.91) | 11 (12.64) | 17 (22.08) | 18 (6.29) | <0.001 |
| Not Comfortable Speaking English | 234 (29.25) | 131 (42.95) | 41 (36.94) | 34 (37.78) | 28 (9.52) | <0.001 |
| **Sex Work Factors** |  |  |  |  |  |  |
| Years Engaged in Sex Work^1^ | 8 (2, 16) | 5 (1, 13) | 6 (2, 11) | 7 (2, 15) | 12 (5, 18) | <0.001 |
| Primary Place Soliciting Clients |  |  |  |  |  | <0.001 |
| Street/public (reference) | 359 (45.33) | 97 (32.12) | 39 (35.14) | 32 (36.36) | 191 (65.64) |  |
| Indoor (formal or informal) | 266 (33.59) | 150 (49.67) | 49 (44.14) | 33 (37.50) | 34 (11.68) |  |
| Independent | 167 (21.09) | 55 (18.21) | 23 (20.72) | 23 (26.14) | 66 (22.68) |  |
| Primary Place Servicing Clients |  |  |  |  |  | <0.001 |
| Street/public (reference) | 312 (39.75) | 84 (28.09) | 35 (31.53) | 33 (37.08) | 160 (55.94) |  |
| Indoor (formal or informal) | 198 (25.22) | 62 (20.74) | 26 (23.42) | 19 (21.35) | 91 (31.82) |  |
| Independent | 275 (35.03) | 153 (51.17) | 50 (45.05) | 37 (41.57) | 35 (12.24) |  |
| Inconsistent Condom use for Vaginal/anal Sex with Clients | 96 (12.06) | 31 (10.16) | 10 (9.09) | 11 (12.50) | 44 (15.02) | 0.220 |
| **Sociostructural Factors** |  |  |  |  |  |  |
| Any Injection Drug Use | 316 (39.40) | 81 (26.47) | 42 (37.84) | 35 (38.89) | 158 (53.56) | <0.001 |
| Any Unstable Housing | 568 (70.91) | 188 (61.24) | 66 (59.46) | 63 (70.00) | 251 (85.67) | <0.001 |
| High School Graduate | 450 (56.11) | 194 (63.40) | 68 (61.26) | 55 (61.11) | 133 (45.08) | <0.001 |
| *Note*. Categorical data summarized as frequencies and percentages. For binary indicators, only affirmative responses are shown.   1. Numeric variable summarized as median and IQR. 2. For numeric variables, comparisons made using the Kruskal-Wallis rank sum test. For categorical variables, comparisons made using Pearson’s Chi-Square test or Fisher’s exact test with simulated p-values using 2,000 replicates (for small sample sizes). | | | | | | |

**SUPPLEMENT B - Analysis of Frequency and Type of Contraception Access Barriers**

Figure B.1: Frequency Distribution of Contraceptive Barriers Experienced in Last 6 Months at Any Given Study Visit, 2010- 2022
